# Comparative Outcomes of Teacher Coaching Versus Parent‐Implemented Intervention for Preschoolers at Risk of Developmental Delay in Thailand

**DOI:** 10.1155/nrp/6653365

**Published:** 2026-03-10

**Authors:** Somsiri Rungamornrat, Yuwadee Pongsaranuntakul, Jinnaphat Sangngam, Apawan Nookong

**Affiliations:** ^1^ Department of Pediatric Nursing, Faculty of Nursing, Mahidol University, Bangkok, Thailand, mahidol.ac.th

**Keywords:** community daycare centers, developmental delays, developmental surveillance and promotion manual (DSPM), early childhood development, low- and middle-income countries (LMICs), parent-implemented intervention, practice-based coaching

## Abstract

**Background:**

Early childhood developmental delays are a major concern in low‐ and middle‐income countries (LMICs), where gaps often exist between developmental surveillance and follow‐up support. In Thailand, the Developmental Surveillance and Promotion Manual (DSPM) provides a national framework for screening and promotion; however, practical models for integrating DSPM guidance into daily classroom and home routines in community daycare settings remain limited.

**Aim:**

To compare two brief nurse‐facilitated implementation models—teacher coaching using practice‐based coaching (PBC) and a parent‐implemented intervention (PII)—and examine their effects on adult practices and short‐term developmental outcomes in preschool children at risk of delay.

**Methods:**

A quasi‐experimental, two‐group pretest–posttest study was conducted in two community daycare centers. Forty‐nine at‐risk children and primary caregivers were enrolled (PBC: 26 dyads; PII: 23 dyads), along with 17 teachers (PBC: 8; PII: 9). Each center implemented one model over a 4‐week intervention period with follow‐up assessment approximately 4 weeks later. Outcomes included teacher developmental knowledge, teacher and caregiver developmental‐promotion behaviors, and child pass/fail status across five DSPM domains. Continuous outcomes were analyzed using 2 × 2 mixed‐design repeated‐measures ANOVA; within‐center domain changes used exact McNemar’s tests.

**Results:**

Teacher knowledge improved over time, and the magnitude of improvement differed between models (group‐by‐time interaction), with larger gains in the PII center than the PBC center. Teacher and caregiver developmental‐promotion behaviors improved significantly from pre to post (main effects of time), while group‐by‐time interactions were not significant, indicating comparable behavioral gains across pathways. Within‐center DSPM analyses suggested short‐term reductions in delayed status in selected domains in both centers.

**Conclusion:**

Both PBC and PII were feasible in community daycare settings and associated with meaningful short‐term improvements in adult developmental‐promotion practices and selected DSPM domains. Child domain patterns should be interpreted descriptively because DSPM changes were analyzed within centers rather than as between‐model contrasts to inform service planning and scale‐up.

**Trial Registration:** Thai Clinical Trials Registry (TCTR): TCTR20250106003

## 1. Introduction

Early childhood developmental delays remain a major global concern, particularly in low‐ and middle‐income countries (LMICs), where many children are at risk of not reaching their developmental potential due to poverty, undernutrition, limited early learning opportunities, and exposure to stress [1, 2]. Inequalities in early childhood—driven by cumulative biological and environmental risks—have long‐term implications for schooling, productivity, and adult health [3]. In response, global initiatives, such as the Lancet Nurturing Care series and the Nurturing Care Framework, emphasize that young children require integrated support across health, nutrition, security and safety, responsive caregiving, and early learning to thrive [4, 5].

Beyond expanding access to services, recent research highlights the importance of process quality—the everyday, emotionally supportive, and language‐rich interactions between adults and children—as a proximal driver of gains in language, socioemotional skills, and school readiness [6, 7]. Studies in early childhood and preprimary settings show that higher quality teacher–child interactions and developmentally appropriate activities are associated with better child outcomes, even in resource‐constrained environments [7, 8]. At the same time, reviews of childcare provision in LMICs indicate that while coverage is expanding, the quality of interactions and the implementation support available to frontline staff remain highly variable [9]. This underscores the need for feasible strategies to strengthen adult–child processes in the settings where young children spend most of their time, particularly community daycare centers and homes serving at‐risk children [7–9].

Thailand has implemented a universal developmental surveillance and promotion system for children aged 0–5 years through the Developmental Surveillance and Promotion Manual (DSPM), which provides age‐anchored milestones across gross motor, fine motor, receptive language, expressive language, and personal–social domains, along with concrete suggestions for daily developmental‐promotion activities [10, 11]. Evaluations of Thailand’s national screening program demonstrate that DSPM can be implemented at scale with high coverage, but gaps remain in follow‐up and consistency in translating screening results into sustained support for families and children, particularly those relying on community daycare centers [11, 12]. Similar concerns about fragmented follow‐up and variable quality of developmental support have been reported in broader reviews of early childhood systems in Thailand and other LMICs [13, 14].

Evidence from nurse‐led early childhood and home‐visiting programs suggests that nurses can play a pivotal role in coordinating developmental surveillance and early intervention linking screening results to parent coaching, promoting responsive caregiving, and strengthening connections between health services and early childhood care settings [15–17]. These findings provide the rationale for testing nurse‐facilitated models that connect DSPM‐identified risks with practical, ongoing support for both teachers and parents in the contexts where children spend their time.

Two promising, practice‐oriented strategies may help bridge this gap in ways compatible with Thai primary care and daycare systems. The first is teacher coaching, operationalized as practice‐based coaching (PBC), which employs cyclical processes of shared goal setting, focused observation in natural routines, and structured feedback (including modeling or coteaching as needed) to enhance teachers’ use of evidence‐based practices [8, 18]. Meta‐analyses of rigorous teacher coaching trials from prekindergarten through secondary school report moderate improvements in instructional practice and smaller but meaningful gains in student achievement, suggesting that well‐designed coaching can shift adult behavior and child learning outcomes at scale [18].

The second strategy is parent‐implemented intervention (PII), which combines brief didactic input with live or video modeling, guided rehearsal, immediate feedback, and daily practice embedded in home routines. Meta‐analyses indicate that such interventions improve caregiver responsiveness and children’s expressive and receptive language, particularly when strategies are practiced during play and familiar activities and when coaching components (modeling plus feedback) are included [19, 20]. These practice packages align with contemporary recommendations in early intervention and early childhood special education, emphasizing observable instructional strategies, high‐quality adult–child interactions, and family capacity‐building supports that can be embedded into everyday routines to enhance children’s participation and learning outcomes [21, 22].

From a theoretical perspective, both teacher‐ and parent‐focused models can be understood through Bandura’s social‐learning and self‐efficacy theory, which posits that modeling, guided practice, and performance feedback build self‐efficacy and support behavior change [23], and through Bronfenbrenner’s bioecological model, which situates child development within nested systems and highlights mesosystem linkages between home and early childhood settings [24]. When teacher coaching in daycare and parent‐implemented strategies in the home are aligned around shared developmental goals, they can reinforce each other across contexts, strengthening the proximal processes that drive child development [18, 19, 23]. In Thailand, nurses working in community and primary care services are well positioned to act as boundary spanners between health and education using DSPM to identify at‐risk children, coaching daycare teachers, and equipping parents with practical strategies to support development at home [10, 11].

Building on this background, the present quasi‐experimental study compares two nurse‐facilitated implementation models in community daycare centers serving preschool children at risk of developmental delay after DSPM screening: (1) a teacher coaching model based on PBC cycles with daycare teachers and (2) a PII model in which nurses coach parents to embed DSPM‐consistent developmental‐promotion activities in home routines. Shared outcomes included teachers’ knowledge, teacher and caregiver developmental‐promoting behaviors, and children’s short‐term progress toward age‐expected DSPM milestones. The study aims to generate decision‐ready evidence from a pragmatic comparison of two implementation pathways for Thailand’s community daycare system and similar LMIC contexts. Specifically, the study compares the short‐term pre–post changes associated with these two models on (a) teachers’ developmental knowledge, (b) teacher and caregiver developmental‐promoting behaviors, and (c) children’s short‐term progress toward age‐expected DSPM milestones.

## 2. Methods

### 2.1. Study Design

This quasi‐experimental, two‐arm, site‐assigned study compared two implementation models for promoting development among preschool children at risk: teacher coaching (PBC) and PII. The models were implemented at two public community daycare centers, with each center assigned to one intervention arm for pragmatic and operational reasons (Center 1 = PBC; Center 2 = PII). Throughout this paper, arm refers to the intervention condition and center to the implementation site. The design reflects real‐world constraints in LMIC settings with limited access to specialized developmental services and caregiver training.

### 2.2. Participants and Setting

Two public community daycare centers in Bangkok were purposely selected from facilities rated “good” based on the Bangkok Metropolitan Administration’s internal quality assessment (staff–child ratio, physical environment, and health‐promotion practices). We prioritized smaller centers with ≤ 10 classroom teachers to enhance feasibility and support intervention fidelity within the 4‐week implementation period. We also sought sites that were as comparable as possible in staffing profile (including at least one early childhood bachelor‐trained teacher), population served (low‐ to middle‐income families), implementation of Thailand’s national early childhood curriculum, and routine six‐monthly child health monitoring by public health staff. Eligible centers were contacted by telephone to assess willingness to participate; of those approached, two volunteer centers agreed to join and were enrolled. For clarity, the sites are referred to as the PBC center and the PII center.

Teachers: All classroom teachers at the participating centers were invited to join. Seventeen teachers provided written informed consent (PBC: 8; PII: 9). Most were female and held at least a diploma or bachelor’s degree in early childhood or related fields.

Children: Children aged 2–5 years attending the two centers were screened using the DSPM. Those classified as “at risk” in at least one developmental domain were eligible. From center enrollments of approximately 80 and 95 children, 49 at‐risk children were enrolled (PBC: 26; PII: 23). Exclusion criteria included severe developmental disability, congenital anomalies, or medical conditions requiring specialized care.

Parents/Caregivers: One primary caregiver per enrolled child (usually the mother) was invited to participate and complete caregiver questionnaires. All 49 caregivers consented (PBC: 26; PII: 23).

### 2.3. Measurements

Teacher Knowledge on Early Childhood Development (30 Items; Investigator‐Developed, DSPM‐Aligned): A Thai‐language, investigator‐developed instrument assessed teachers’ knowledge of general developmental principles and age‐anchored milestones across the five DSPM domains. Items are judged true/false/uncertain and scored 1 for correct and 0 for incorrect/uncertain responses (total 0–30; higher scores indicate greater knowledge). Sample items include the following: “Children of the same age always have identical developmental abilities. (True/False/Uncertain)” (correct = false); “A typically developing 2‐3‐year‐old can pedal a two‐wheeled bicycle independently. (True/False/Uncertain)” (correct = false); and “Free play, combined with supportive adult interaction, is one important way to promote child development. (True/False/Uncertain)” (correct = true). Item content and wording were derived directly from Thailand’s DSPM guidance and widely taught milestone benchmarks [10]. Content validity was established by a three‐member expert panel (pediatric nursing faculty and pediatricians), with a content validity index (CVI) of 0.99. In a pilot sample of 20 teachers, internal consistency reliability was Cronbach’s *α* = 0.92.

Child Development Promotion Behavior for Teacher and Caregiver (CDPB‐20; 20 Items; Investigator‐Constructed; DSPM‐Derived; Common Item Pool for Teachers and Caregivers): A 20‐item Thai checklist quantified the frequency of DSPM‐recommended developmental‐promotion behaviors across all domains (gross motor, fine motor, language, and personal–social). The instrument retained the same name, number of items, and core content for both respondent groups; only the context‐anchoring instructions were adapted to reflect the setting (classroom vs. home) while preserving identical item wording. The same items were administered to teachers and caregivers, preceded by brief context‐anchoring instructions to ensure ecological validity. For example, Teachers (PBC Arm): “Please rate how often these practices occur in your classroom during typical routines.” Caregivers (PII Arm): “Please rate how often these practices occur at home during daily routines.” Responses use a 4‐point frequency scale (3 = frequently, 2 = sometimes, 1 = rarely, and 0 = never); total scores range from 0 to 60, with higher scores indicating more frequent use of age‐appropriate promotion behaviors. Sample items (context‐neutral wording) include the following: “You prepare space and organize activities that allow children to run, climb, and practice balance (e.g., standing on one leg/walking on a balance board, two‐foot and one‐foot jumping, jumping over objects, kicking and throwing balls)”; “You organize art activities such as bead threading, coloring, playdough modeling, folding, cutting and pasting paper, drawing geometric shapes, or drawing a person with body parts”; “You provide opportunities for children to learn and use language, such as giving multi‐step instructions, asking children to express feelings/needs, share opinions, and explain meanings of things around them”; and “You train children to develop basic self‐help skills in daily routines (e.g., brushing teeth, dressing, and clearing dishes).” Designated reverse‐scored items were recoded before summation. Items were derived from age‐anchored DSPM promotion guidance and reviewed by the same expert panel as above, yielding CVI = 0.98. In a pilot sample of 20 caregivers, Cronbach’s *α* was 0.87.

Child Developmental Progress Record (DSPM‐Aligned; Five Domains): Child outcomes were operationalized with a DSPM‐aligned record covering gross motor, fine motor, receptive language, expressive language, and personal–social domains. For each child, age‐appropriate DSPM items were administered, and each domain was classified as pass (age‐appropriate) or fail (delayed). We examined pre–post changes for each domain separately and did not compute a composite or total score. This pass/fail schema follows DSPM follow‐up guidance and supports paired categorical analyses using the exact McNemar test, as detailed in the Statistical Analysis section.

Scale Sources and Rationale: All instruments were investigator‐developed or constructed to align directly with Thailand’s DSPM framework and terminology for developmental surveillance and promotion [10]. Content validity was established through expert review (CVI), and internal consistency was assessed using Cronbach’s *α* in pilot samples.

Administration and Scoring: Teachers completed the Knowledge scale and the CDPB‐20 (classroom context); primary caregivers completed the CDPB‐20 (home context). For all scales, higher totals reflect greater knowledge or more frequent use of age‐appropriate promotion behaviors. All measures were administered in Thai immediately before (pre) and after (post) the 4‐week intervention period.

### 2.4. Procedures and Data Collection

This study was conducted over approximately 8–10 weeks in three sequential phases: (1) pre‐intervention screening and baseline assessment; (2) a 4‐week intervention phase; and (3) postintervention follow‐up reassessment. The procedures and data collection schedule for both study arms are summarized in Table 1.

**TABLE 1:** Study phases and procedures for the PBC and PII intervention arms.

Phase	Common procedures (both arms)	PBC arm	PII arm
Pre‐intervention (screening and baseline)	‐ DSPM screening by pediatric nurses for all children aged 2–5 years in each center ‐ Identification of “at‐risk” children; informed consent from teachers and primary caregivers ‐ Baseline Assessment: Teacher Knowledge scale, CDPB‐20 (teacher and caregiver), and DSPM status in 5 domains		

Intervention (4 weeks)		‐ Nurse‐led DSPM workshop for teachers (DSPM domains, interpretation of at‐risk results, and classroom‐based development promotion). ‐ DSPM‐informed class goal setting (2‐3 class goals based on children’s DSPM profiles) and integration into weekly routine plans. ‐ Weekly PBC cycles (goal review/action planning, in‐routine observation, and immediate feedback with modeling/coteaching as needed). Center‐based reinforcement through every 2 weeks peer reflection and coaching logs.	‐ Nurse‐led caregiver workshop (3 h) (DSPM results, rationale, and demonstrations of home‐based development activities) ‐ Individualized activity selection (2‐3 DSPM‐consistent activities embedded in daily routines). ‐ In vivo coaching contact through at least one home visit; when not feasible, coaching provided at daycare drop‐off/pick‐up. ‐ Remote reinforcement via fortnightly video clips/reminder messages and caregiver self‐monitoring checklist

Postintervention follow‐up (4 weeks after intervention)	‐ Reassessment of Teacher Knowledge and CDPB‐20 (teacher and caregiver) ‐ Reassessment of DSPM domain status (5 domains) by nurses per national follow‐up guidance ‐ Referral of children with persistent delay to specialist services		

Key implementation distinctions		Primary Implementer: teachers (supported by nurse coaching). Delivery Context: daycare routines (in vivo). Implementation Support: observation‐ and feedback‐driven teacher practice improvement, reinforced through center‐based peer reflection and documentation.	Primary Implementer: caregivers (supported by nurse/teacher coaching). Delivery Context: home routines (in vivo; supplemented by coaching at drop‐off/pick‐up when home visits not feasible). Implementation Support: routine‐embedded caregiver practice, reinforced through remote prompts and caregiver self‐monitoring.

#### 2.4.1. Pre‐Intervention Phase (Common Procedures)

At each daycare center, all attending children aged 2–5 years were screened by trained pediatric nurses using the age‐appropriate items from the DSPM. Children classified as “at risk” in at least one DSPM domain were invited, together with their primary caregivers, to participate in the study.

After written informed consent was obtained from teachers and caregivers, baseline data were collected as follows. Teachers completed the Teacher Knowledge on Early Childhood Development scale and the CDPB‐20 checklist (classroom context). Primary caregivers completed the CDPB‐20 (home context). For each enrolled child, developmental status (pass/fail) in all five DSPM domains (gross motor, fine motor, receptive language, expressive language, and personal–social) was assessed and recorded according to national DSPM guidance.

All baseline assessments were administered in Thai by research nurses who were not involved in delivering the subsequent intervention sessions.

#### 2.4.2. Intervention Phase (Arm‐specific Procedures; 4 Weeks)

During the 4‐week intervention period, each center implemented one implementation model: a teacher coaching model in the PBC arm and a PII model in the PII arm. Although both models incorporated DSPM‐aligned developmental targets and skills practice, they differed in the primary implementer, the setting of coaching, and the approach to implementation monitoring. Specifically, PBC provided in vivo coaching to teachers within daycare routines through iterative observation and feedback, whereas PII supported caregivers to implement individualized activities within home routines, with additional in‐person coaching contact and remote reinforcement.

##### 2.4.2.1. Teacher Coaching Model (PBC Arm)

In the teacher coaching (PBC) arm, the focus was on strengthening teachers’ use of development‐promoting practices during everyday classroom routines.

a. Initial workshop and goal setting
 All teachers in the PBC center attended a structured group workshop led by a pediatric nurse. The workshop reviewed DSPM domains, interpretation of “at risk” results, and core principles for embedding developmental‐promotion activities in daily classroom routines. Training hours were tailored to teachers’ educational background (advanced sessions for early childhood graduates and more foundational content for noneducation graduates).
 Using each child’s DSPM profile, the nurse and teachers jointly identified two to three concrete goals per class (e.g., increasing fine‐motor practice opportunities, expanding expressive language turns, and adding predictable personal–social routines) and drafted simple weekly lesson plans that integrated these goals.
b. Practice‐based coaching cycles
 Over the 4 weeks, the nurse conducted repeated coaching cycles with each teacher following a practice‐based coaching structure:
  • Shared Goal Review and Action Planning: Confirming or refining the weekly classroom plan (e.g., adding a recycled‐materials fine‐motor activity, planning a structured storytelling circle, or building turn‐taking games into group play).
  • Focused Observation: Observing the teacher during naturally occurring routines (such as circle time, free play, meal times, or transition periods) using a brief observation checklist aligned with the CDPB‐20 items and DSPM guidance.
  • Feedback and Reflection: Immediately after observation, the nurse provided specific, behavior‐focused feedback, highlighted strengths, modeled strategies or cotaught short segments when needed, and jointly problem‐solved barriers. Teachers then adjusted their plans for the following week.
c. Ongoing support and peer reflection
 Short, every 2 weeks peer discussions were held at the center, facilitated by the nurse. Teachers shared examples of successful activities, challenges with particular children, and adaptations made in their classrooms. A simple coaching log documented goals, strategies implemented, and teachers’ reflections on children’s responses.

##### 2.4.2.2. PII Model (PII Arm)

In the PII arm, the focus was on equipping caregivers to embed DSPM‐consistent activities into everyday home routines, with nurses and teachers acting as coaches.

a. Group DSPM workshop for caregivers
 Caregivers attended a 3‐h, nurse‐led group session that covered DSPM domains and interpretation of their child’s “at risk” results, as well as examples of age‐appropriate activities that can be integrated into daily life (e.g., storytelling before bedtime, recycled‐materials fine‐motor play, simple turn‐taking games, and small household helping tasks, such as setting the table). The workshop combined brief presentations, visual materials, live or video demonstrations, and role‐play, allowing caregivers to rehearse strategies, such as labeling and expansion of child utterances, scaffolding fine‐motor tasks, and encouraging cooperative play.
b. Individualized home‐routine coaching and home visits
 Based on each child’s DSPM profile, the nurse and daycare teacher helped caregivers select two to three specific activities to practice every day within familiar routines (e.g., telling short stories during bath time or before sleep, making simple toys from recycled materials to encourage grasping and manipulation, and involving the child in small household chores to build gross motor and personal–social skills).
 Teachers and nurses conducted at least one home visit when feasible to demonstrate the target activities in the child’s actual home environment, observe caregiver–child interaction, and provide immediate feedback and encouragement. When home visits were not possible, demonstrations and coaching were provided at the daycare center during arrival or pick‐up times.
c. Remote follow‐up and self‐monitoring
 Throughout the 4‐week period, nurses sent brief educational video clips and reminder messages (e.g., via smartphone applications) approximately every 2 weeks. These materials reinforced key strategies linked to the child’s DSPM domains and provided new examples of simple games or routines. Caregivers were invited to mark how often they used the suggested activities on a one‐page checklist, which was later discussed during follow‐up contact.

#### 2.4.3. Postintervention Follow‐Up (Common Procedures)

Approximately 4 weeks after completion of the intervention phase, all baseline measures were re‐administered in both arms. Teachers again completed the Teacher Knowledge scale and the CDPB‐20 (classroom context), and primary caregivers repeated the CDPB‐20 (home context). Children’s developmental status in each DSPM domain was reassessed by nurses following national DSPM follow‐up recommendations, which advise repeat assessment about 1 month after initial identification of suspected delay.

Children who continued to show delay in one or more DSPM domains after the follow‐up assessment were referred to appropriate specialist services (e.g., pediatric or developmental clinics) according to routine care pathways. Pre–post DSPM domain status was assessed as paired data within the same child.

### 2.5. Ethical Consideration

Ethical approval was obtained from the Research Ethics Committee of the Faculty of Nursing, Mahidol University (IRB‐NS2023/744.2601). Written informed consent was obtained from all participating teachers and from the primary caregivers of all enrolled children in both study arms before any data collection. Each adult participant received a participant information sheet and consent form describing the study aims, procedures, potential benefits and risks, and measures to protect confidentiality. Caregivers provided permission for their child’s participation. Participants were informed that participation was voluntary and that they could withdraw from the study at any time without any consequences for their employment or access to services. In this study, no participants withdrew. All procedures complied with institutional ethical standards and were conducted in accordance with the principles of the Declaration of Helsinki.

### 2.6. Statistical Analyses

Continuous outcomes (Teacher Knowledge and CDPB‐20 total scores for teachers and caregivers) were assessed at two time points (pre‐ and postintervention). Intervention effects were examined using a 2 (group: PBC vs PII) × 2 (time: pre vs post) mixed‐design repeated‐measures ANOVA, with time specified as the within‐subject factor and group as the between‐subject factor. Main effects of time and group and the group × time interaction were reported. Effect sizes were expressed as partial eta squared (*η* *p* ^2^), calculated as SS_effect/(SS_effect + SS_error) for each effect (time, group, and group × time), and interpreted using conventional benchmarks (approximately 0.01 small, 0.06 medium, and 0.14 large) [25], recognizing that practical relevance is context‐dependent. Where relevant, estimated marginal means were used to describe overall pre–post change. Model assumptions were evaluated using standard diagnostics (e.g., Levene’s test for homogeneity of variance); with two time points, sphericity was not applicable.

For categorical child DSPM outcomes (domain status: age‐appropriate vs delayed), within‐group pre–post changes were analyzed using the exact McNemar test for paired nominal data. All statistical tests were two‐sided with *α* = 0.05. Analyses were conducted using SPSS (IBM SPSS Statistics).

## 3. Results

### 3.1. Sample

Seventeen teachers were included (PBC, *n* = 8; PII, *n* = 9). Forty‐nine at‐risk children and their primary caregivers completed pre‐ and postassessments (PBC, *n* = 26 dyads; PII, *n* = 23 dyads).

### 3.2. Teacher Knowledge of Early‐Childhood Development

Both groups started with comparable baseline knowledge (Table 2). A 2 (group) × 2 (time) mixed‐design repeated‐measures ANOVA showed a significant main effect of time, indicating an overall increase in Teacher Knowledge from pre‐ to postintervention, *F* (1, 15) = 11.44, *p* = 0.004, *η* *p* ^2^=0.433. The group × time interaction was significant, *F* (1, 15) = 4.72, *p* = 0.046, *η* *p* ^2^=0.239, indicating that knowledge gains differed by model. Descriptively, the PII group increased from 23.88 ± 1.16 to 27.33 ± 2.18, whereas the PBC group showed a smaller increase from 23.75 ± 0.70 to 24.50 ± 1.69. A significant between‐group main effect was also observed, *F* (1, 15) = 11.93, *p* = 0.004, *η* *p* ^2^=0.443 (Table 2).

**TABLE 2:** Teacher Knowledge (DSPM‐aligned, 0–30): pre–post scores by model and mixed‐design repeated‐measures ANOVA.

**Panel A descriptive statistics**			
**Model (*n*)**	**Pre mean ± SD**	**Post mean ± SD**	**Δ mean**

PBC (*n* = 8)	23.75 ± 0.70	24.50 ± 1.69	+0.75
PII (*n* = 9)	23.88 ± 1.16	27.33 ± 2.17	+3.45

**Panel B mixed-design repeated-measures ANOVA (2 group × 2 time)**			
**Effect**	***F* (df1, df2)**	**p** **value**	**Partial** **η** **p** ^2^

Time (pre vs post)	*F* (1, 15) = 11.44	0.004	0.433
Group (PBC vs PII)	*F* (1, 15) = 11.93	0.004	0.443
Time × group	*F* (1, 15) = 4.72	0.046	0.239

*Note:* Mixed‐design repeated‐measures ANOVA tested time (within‐subject), group (between‐subject), and their interaction. Higher scores indicate greater knowledge.

### 3.3. Teacher Behavior in Child Development Promotion

Teacher developmental‐promotion behaviors improved from pre to post in both models (Table 3). Mixed‐design repeated‐measures ANOVA indicated a significant main effect of time, *F* (1, 15) = 40.39, *p* < 0.001, *η* *p* ^2^=0.729, reflecting increased CDPB‐20 scores across groups. The group × time interaction was not significant, *F* (1, 15) = 0.08, *p* = 0.786, *η* *p* ^2^=0.005, suggesting comparable improvements between PBC and PII. The between‐group main effect was not statistically significant, *F* (1, 15) = 3.81, *p* = 0.070, *η* *p* ^2^=0.202 (Table 3).

**TABLE 3:** Teacher Child Development Promotion Behavior (CDPB‐20, 0–60): pre–post scores by model and mixed‐design repeated‐measures ANOVA.

**Panel A descriptive statistics**			
**Model (*n*)**	**Pre mean ± SD**	**Post mean ± SD**	**Δ mean**

PBC (*n* = 8)	43.38 ± 2.62	48.88 ± 2.70	+5.50
PII (*n* = 9)	45.56 ± 2.19	51.56 ± 4.53	+6.00

**Panel B mixed-design repeated-measures ANOVA (2 group × 2 time)**			
**Effect**	***F* (df1, df2)**	**p** **value**	**Partial** **η** **p** ^2^

Time (pre vs post)	*F* (1, 15) = 40.39	< 0.001	0.729
Group (PBC vs PII)	*F* (1, 15) = 3.81	0.070	0.202
Time × group	*F* (1, 15) = 0.08	0.786	0.005

*Note:* Higher scores indicate more frequent teacher use of DSPM‐aligned developmental‐promotion behaviors.

### 3.4. Caregiver Behavior in Child Development Promotion

Caregiver developmental‐promotion behaviors also improved over time in both models (Table 4). Mixed‐design repeated‐measures ANOVA demonstrated a significant main effect of time, *F* (1, 47) = 23.48, *p* < 0.001, *η* *p* ^2^=0.333. The group × time interaction was not significant, *F* (1, 47) = 0.46, *p* = 0.499, *η* *p* ^2^=0.010, indicating no evidence that one model produced greater caregiver behavior change than the other. The between‐group main effect was not significant, *F* (1, 47) = 0.00, *p* = 0.962, *η* *p* ^2^=0.000 (Table 4).

**TABLE 4:** Caregiver Child Development Promotion Behavior (CDPB‐20, 0–60): pre–post scores by model and mixed‐design repeated‐measures ANOVA.

**Panel A descriptive statistics**			
**Model (*n*)**	**Pre mean ± SD**	**Post mean ± SD**	**Δ mean**

PBC (*n* = 26)	35.15 ± 8.48	41.50 ± 10.60	+6.35
PII (*n* = 23)	35.83 ± 7.92	40.61 ± 8.21	+4.78

**Panel B mixed-design repeated-measures ANOVA (2 group × 2 time)**			
**Effect**	***F* (df1, df2)**	**p** **value**	**Partial** **η** **p** ^2^

Time (pre vs post)	*F* (1, 47) = 23.48	< 0.001	0.333
Group (PBC vs PII)	*F* (1, 47) = 0.00	0.962	0.000
Time × group	*F* (1, 47) = 0.46	0.499	0.010

*Note:* Higher scores indicate more frequent caregiver use of DSPM‐aligned developmental‐promotion behaviors.

### 3.5. Child Developmental Status by DSPM Domain (Within‐Group Change)

Within‐group analyses using exact McNemar tests showed that in the PBC center (*n* = 26), the proportion of children classified as delayed decreased significantly in fine‐motor, expressive language, and personal–social domains (all *p* < 0.05), with a borderline improvement in receptive language (*p* = 0.063) and a nonsignificant change in gross motor (*p* = 0.125). In the PII center (*n* = 23), significant reductions in delay were observed for gross motor and personal–social (both *p* = 0.031), whereas fine motor and receptive language showed no change (no discordant pairs), and expressive language exhibited a small, nonsignificant improvement (*p* = 0.500) as shown in Table 5.

**TABLE 5:** The comparison of child DSPM domain status within group (McNemar exact), PBC (*n* = 26), and child DSPM domain status within group (McNemar exact), PII (*n* = 23).

Domain	Model	Pre: delayed (*n*)	Post: delayed (*n*)	Discordant pairs	McNemar exact *p*
GM	PBC	4	0	4/0	0.125
	PII	6	0	6/0	0.031

FM	PBC	12	3	9/0	0.004
	PII	9	6	3/0	0.250

RL	PBC	9	4	5/0	0.063
	PII	9	7	2/0	0.500

EL	PBC	15	5	10/0	0.002
	PII	12	10	2/0	0.500

PS	PBC	7	0	7/0	0.016
	PII	6	0	6/0	0.031

## 4. Discussion

This quasi‐experimental study compared two short, theory‐based models—teacher coaching (PBC) and PII—for linking DSPM‐based screening to follow‐up support in community daycare centers. Overall, both models were associated with positive short‐term change: Teachers reported more frequent development‐promoting practices, caregivers increased the frequency of stimulation at home, and within each center, the proportion of children classified as delayed decreased in selected DSPM domains over approximately 1 month of follow‐up, corresponding to the national DSPM reassessment window [10]. These findings align with broader literature indicating that even relatively low‐intensity supports targeting daily adult–child interactions can improve proximal processes and near‐term developmental outcomes, including resource‐constrained early childhood settings [8, 18, 19].

An unexpected finding emerged regarding Teacher Knowledge. Although we anticipated the PBC arm would show the largest gains, Teacher Knowledge improved over time, and the magnitude of improvement differed between models, with larger gains in the PII arm than in the PBC arm. One plausible explanation is that the PII package may have placed greater emphasis on explicit content preparation (e.g., structured review of milestones and preparation for caregiver sessions), requiring teachers to organize and verbalize developmental concepts clearly. This pattern aligns with evidence that parent‐implemented programs combining brief instruction with practice and feedback can enhance adult understanding of developmental strategies, especially when adults are expected to “teach back” or model skills at home [26–28]. Additionally, the narrow baseline knowledge spread in the PBC center may have limited detectable change. Both arms included modeling, guided practice, and feedback which are key mechanisms of mastery experience and self‐efficacy, yet the balance between content teaching and practice coaching differed. In contexts where teachers already hold moderate knowledge, structured content designed to support explanation to caregivers may consolidate conceptual understanding.

In contrast, adult caregiving behaviors improved markedly and similarly across both arms. Teachers reported higher frequencies of development‐promoting practices, and caregivers increased use of DSPM‐aligned activities. Pre–post improvements in both teacher and caregiver behaviors suggest that brief nurse‐facilitated supports can strengthen short‐term day‐to‐day developmental stimulation in daycare and home routines. These results align with evidence that coaching‐style strategies, including goal setting, in situ observation, and feedback, reliably shift adult practices when embedded in daily routines [8, 18, 26–28]. From a social‐learning perspective, both PBC and PII provided opportunities for mastery experiences, vicarious learning, and supportive feedback, which are central to building self‐efficacy and sustaining behavior change [23]. For program planners, these findings suggest that both teacher‐ and caregiver‐focused entry points can effectively influence proximal environments, and choice of approach may depend more on staffing, feasibility, and service priorities than on short‐term behavioral impact.

Child‐level DSPM outcomes showed domain‐specific patterns consistent with the operational focus of each model. These domain‐specific patterns should be interpreted descriptively because child DSPM changes were examined within centers rather than as between‐model contrasts. In the PBC center, significant reductions in delay were observed in fine motor, expressive language, and personal–social domains, with borderline improvement in receptive language, areas often responsive to structured small‐group classroom activities. In the PII center, significant reductions were found in gross motor and personal–social domains, influenced by home routines, such as outdoor play, simple chores, and family interactions. The absence of significant change in some domains (e.g., receptive language and fine motor in PII) highlights that not all developmental areas shift within a short follow‐up, even when adult behaviors improve. Meta‐analyses indicate that gains are sensitive to dosage, content focus, and strategy–skill match [26, 29].

Both arms produced meaningful improvements in caregiver behaviors, demonstrating that caregivers benefit when guidance is concrete, feasible, and supported by simple monitoring and follow‐up, regardless of whether the intervention is teacher‐ or parent‐focused. This finding is encouraging within the Thai DSPM system, indicating that screening can be linked to actionable follow‐up through either pathway.

From a time and sustainability perspective, the 1‐month intervention cycle aligns with national DSPM follow‐up guidance. This duration appeared sufficient to detect short‐term movement from “delayed” to “age‐expected” status in selected domains, although longer or repeated cycles may produce stronger and more stable gains, particularly for children with multiple delays or complex social risks [26, 27, 29].

Overall, this study contributes evidence on how to move from “screening to support” in LMIC daycare systems. Both PBC and PII models were feasible within Thai community centers and associated with meaningful short‐term changes in adult practices and selected child outcomes. For nursing and allied health professionals, these results highlight practical roles in coaching teachers, facilitating caregivers, and linking DSPM screening to structured follow‐up. While sample size, short follow‐up, and absence of a nonintervention control group limit causal inference, findings suggest that nurse‐led, brief, structured supports can make DSPM actionable in daily daycare and home routines.

### 4.1. Limitations

1. Design and Sample Size: The quasi‐experimental design involved only two daycare centers (one per model) with a small sample of teachers and caregiver–child dyads. Allocation by center may have introduced unmeasured confounding, and between‐model inference is limited because each model was implemented in a single center. Results should be interpreted as preliminary and hypothesis‐generating.
2. Short Follow‐up: Outcomes were assessed over a short baseline‐to‐follow‐up period. This timeframe does not allow conclusions about maintenance, decay, or longer‐term effects on school readiness.
3. Self‐reported Behaviors: Teacher and caregiver behaviors were self‐reported, which may be affected by recall bias and social desirability. Independent observational verification was not conducted; future studies should incorporate observational tools.

### 4.2. Conclusion

Both teacher coaching (PBC) and PII were associated with meaningful short‐term improvements in adult practices and selected child outcomes in Thai community daycare centers. Teachers and caregivers in both arms reported more frequent development‐promoting behaviors, and fewer children were classified as delayed in specific DSPM domains.

The magnitude of pre–post improvement in Teacher Knowledge differed between models, with larger gains in the PII arm than in the PBC arm. Behavioral changes were substantial and comparable across arms. This suggests that both teacher‐ and caregiver‐focused supports can effectively influence proximal environments when grounded in social‐learning principles, and that explicit didactic components may enhance teachers’ conceptual understanding. Overall, nurse‐facilitated, low‐dose PBC and PII models are feasible and help make DSPM screening actionable by linking identification to structured follow‐up in classroom and home contexts.

### 4.3. Recommendations

Future work should build on these findings in three directions. First, program implementation could be strengthened by integrating either teacher coaching or PII as a routine follow‐up step after DSPM screening in community daycare centers, with nurses acting as facilitators and coaches. Service planners may choose the primary pathway—teacher‐focused, caregiver‐focused, or a combination—based on staffing, existing collaborations, and local priorities, while preserving key components, such as brief cycles of goal setting, modeling, guided practice, and simple monitoring tools.

Second, research designs should be extended to include larger samples, more centers, and longer follow‐up. Cluster‐randomized or stepped‐wedge trials comparing PBC, PII, and hybrid or sequential models over 6–12 months would enable more robust estimation of time effects and group × time differences across outcomes, while accounting for center‐level clustering. Incorporating independent observational measures of adult–child interaction quality, alongside DSPM and more sensitive developmental assessments, would allow a clearer test of how changes in process quality translate into child outcomes.

Third, policy and training efforts should consider the central role of nurses at the interface between health and early education. Developing scalable training packages that equip nurses to coach teachers and caregivers, interpret DSPM results, and codesign routine‐based stimulation activities could help reduce the gap between “screening” and “support” in Thailand’s national developmental surveillance system and offer a practical model for other low‐ and middle‐income settings facing similar implementation challenges.

## Author Contributions

Somsiri Rungamornrat and Yuwadee Pongsaranuntakul jointly led the study. They were responsible for conceptualization, study design, development of the PBC and PII intervention materials and instruments, coordination with the daycare centers, data analysis and interpretation, and drafting, reviewing, and editing the manuscript. Jinnaphat Sangngam contributed to methodology refinement, coordinated fieldwork, led data collection and management, and assisted with interpretation of the findings. Apawan Nookong provided overall supervision, contributed to the conceptualization and study design, ensured alignment with DSPM policy and service context, and critically reviewed and edited the manuscript for important intellectual content.

## Funding

This project was funded by the Educational Equity in Thailand (EFF).

## Disclosure

All authors read and approved the final manuscript.

## Conflicts of Interest

The authors declare no conflicts of interest.

## Data Availability

The data that support the findings of this study are available from the corresponding author upon reasonable request. The complete questionnaires are available from the corresponding author upon reasonable request and are provided for peer‐review and research purposes. Redistribution is not permitted without permission.

## Appendix A Teacher Knowledge on Early Childhood Development Questionnaire

Developed By: Somsiri Rungamornrat, Yuwadee Pongsaranuntakul, Jinnaphat Sangngam, Apawan Nookong, Faculty of Nursing, Mahidol University, Thailand.
Informed By: Thailand’s Developmental Surveillance and Promotion Manual (DSPM) and evidence‐based principles of early childhood developmental promotion.
Target Respondents: Teachers of preschool‐age children.
Administration: Self‐administered; paper or online.
Estimated Completion Time: 20–30 min.
Purpose: To assess teachers’ knowledge of general developmental principles and DSPM‐informed, age‐anchored milestone expectations, including applied interpretation of developmental domains.
Instrument Structure: 30 statements grouped into three sections:
  • General concepts of child development (items 1–12)
  • Age‐anchored statements (DSPM‐aligned; items 13–21)
  • Domain interpretation and applied knowledge (items 22–30)
Response Options: true/false/uncertain.
Scoring: Correct response (as specified in the answer key) = 1; incorrect/uncertain response = 0. Total score range: 0–30 (higher scores indicate greater knowledge).

Sample items (illustrative)

General Concepts:

3. “Children of the same age always have identical developmental abilities. (True/False/Uncertain)” (correct = false).
Age‐anchored (DSPM‐informed):
13. “A typically developing 2‐3‐year‐old can pedal a two‐wheeled bicycle independently. (True/False/Uncertain)” (correct = false).
Applied Knowledge:
29. “Free play, combined with supportive adult interaction, is one important way to promote child development. (True/False/Uncertain)” (correct = true).

## Appendix B Child Development Promotion Behavior for Teacher and Caregiver

Developed By: Somsiri Rungamornrat, Yuwadee Pongsaranuntakul, Jinnaphat Sangngam, Apawan Nookong; Faculty of Nursing, Mahidol University, Thailand.
Informed By: Thailand’s Developmental Surveillance and Promotion Manual (DSPM) and evidence‐based principles of early childhood developmental promotion.
Target Respondents: Teachers of preschool‐age children and primary caregivers of preschool‐age children.
Administration: Self‐administered; paper or online.
Estimated Completion Time: 15–30 min.
Purpose: To quantify the frequency of DSPM‐recommended developmental‐promotion behaviors during typical routines in the classroom (teachers) or home (caregivers).
Instrument Structure: A common 20‐item pool is used for both teachers and caregivers. Items are grouped into four domains:
  • Gross motor development (items 1–5)
  • Fine motor development (items 6–10)
  • Language development (items 11–15)
  • Personal–social/self‐help development (items 16–20)
Context‐Anchoring Instructions: The same item wording is retained for both respondent groups; only the setting in the instructions is adapted to support ecological validity, for example:
  • Teachers: “Please rate how often these practices occur in your classroom during typical routines.”
  • Caregivers: “Please rate how often these practices occur at home during daily routines.”
Response Options and Scoring: A 4‐point frequency scale is used: 3 = frequently, 2 = sometimes, 1 = rarely, 0 = never.
Score Ranges: Each domain score ranges from 0 to 15. Total score ranges from 0 to 60; higher scores indicate more frequent use of age‐appropriate developmental‐promotion behaviors.
Reverse Scoring: Any designated reverse‐scored items (as specified in the full instrument) should be recoded before summation.
Sample Items:
1. You prepare space and organize activities that allow children to run, climb, and practice balance (e.g., standing on one leg/walking on a balance board, two‐foot and one‐foot jumping, jumping over objects, and kicking and throwing balls).
8. You organize art activities, such as bead threading, coloring, playdough modeling, folding, cutting and pasting paper, drawing geometric shapes, or drawing a person with body parts.
11. You provide opportunities for children to learn and use language, such as giving multistep instructions, asking children to express feelings/needs, share opinions, and explain meanings of things around them.
16. You train children to develop basic self‐help skills in daily routines (e.g., brushing teeth, dressing, and clearing dishes).
